# Phenotypic Associations in Resistance to Medically Important Antibiotics Among Highly Resistant Bacteria Isolated from Cambodian Dairy Farms

**DOI:** 10.3390/biology15201871

**Published:** 2026-10-16

**Authors:** Kanan Dim, Sho Nakamura, Soriya Rin, Sophorn Nouv, Sambo Na, Panhavatey Sokhom, Shuichi Matsuyama, Satoshi Ohkura, Witaya Suriyasathaporn

**Affiliations:** 1 Cambodia Campus, Asian Satellite Campuses Institute, Nagoya University, Nagoya 464-8601, Japan; 2 Faculty of Veterinary Medicine, Royal University of Agriculture, Phnom Penh 120501, Cambodia; 3 Laboratory of Animal Production Science, Graduate School of Bioagricultural Sciences, Nagoya University, Nagoya 464-8601, Japan; 4 Department of Animal Health and Veterinary Public Health, General Directorate of Animal Health and Production, Phnom Penh 120603, Cambodia; 5 Faculty of Veterinary Medicine, Chiang Mai University, Chiang Mai 50100, Thailand; 6 Research Center of Producing and Development of Products and Innovations for Animal Health and Production, Chiang Mai University, Chiang Mai 50100, Thailand

**Keywords:** antibiotic resistance, medically important antibiotics, multidrug-resistant bacteria, dairy cattle, milk testing

## Abstract

Antimicrobial resistance (AMR) is recognized by global health authorities as one of the foremost public health and development challenges confronting humanity today, leading the WHO to establish categories of medically important antibiotics for humans and animals. On farms with a high burden of infection, inappropriate antibiotic use, mixed bacterial infections, and environmental contamination with antibiotics from animal sources might accelerate the development of antibiotic resistance, particularly from a One Health Approach. Therefore, this study was conducted to determine correlations in AMR among antibiotics using bacteria isolated from dairy farms with a high burden of intramammary infections. In addition, antibiotic resistance percentages were compared between a smallholder dairy farm and an intensive dairy farm in Cambodia, where the dairy industry has been established for only a few years. Bacterial isolates were collected from two dairy farms: Farm A, a medium-sized local farm with approximately 30 milking cows, and Farm B, a large intensive farm with approximately 480 milking purebred Holstein-Friesian milking cows. All bacteria were tested for antimicrobial susceptibility using a disk diffusion test with 17 antibiotics from nine classes. Classifications based on CLSI interpretive criteria were distinguished from those based on study-defined, extrapolated inhibition-zone thresholds, designated expected AMR (AMR-E). The latter classifications were exploratory and were not validated measures of resistance. The antimicrobials included antibiotics from classes important in human medicine: human-used only (HU), highest-priority critically important antimicrobials (HPCIA), critically important antimicrobials (CIAs), and highly important antimicrobials (HIAs). Fisher’s exact test was used to compare AMR between farms (*p* < 0.05), and Spearman’s correlation adjusted by a Benjamini–Hochberg (Q < 0.05) was used to evaluate associations among antimicrobial susceptibility classifications and between these classifications and multidrug resistance (MDR) status. The results showed that the distribution of bacteria differed between the two farms. For antibiotics used only in humans, no imipenem-resistant Enterobacterales were detected, whereas three Enterobacterales isolates were classified as resistant to meropenem. Differences between farms varied by bacterial group and antimicrobial agent. Many significant correlations in AMR were found among antibiotics, especially those within the same class. Different correlations among antibiotics were found across bacterial groups, including Enterobacterales, staphylococci, enterococci, and streptococci. Phenotypic associations were observed among antimicrobial susceptibility classifications, particularly for agents within the same class. These associations do not establish shared resistance mechanisms or predict treatment outcomes. Findings based on extrapolated thresholds require validation using appropriate susceptibility testing methods.

## Simple Summary

Antibiotic resistance occurs when bacteria survive medicines designed to kill them or stop their growth, threatening both animal and human health. Little is known about this problem on dairy farms in Cambodia, where commercial dairy farming is relatively new. We examined 186 laboratory-grown bacterial samples obtained from milk collected on two Cambodian dairy farms and tested their responses to 17 antibiotics, including medicines important for treating people. We also examined whether bacterial responses to different antibiotics were associated. For some bacterium-antibiotic combinations, established interpretation criteria were unavailable, so exploratory classifications were analyzed separately. Patterns differed between the two farms, and responses to antibiotics within the same class were often associated. These findings support further surveillance, but the exploratory results require confirmation and cannot establish resistance mechanisms or guide treatment choices.

## 1. Introduction

Antimicrobial resistance (AMR) is recognized by global health authorities as one of the foremost public health and development challenges confronting humanity today. This phenomenon is not a speculative future threat; it is already manifesting and undermining the foundational principles of modern medicine [1]. Based on predictive statistical models, an estimated 4.95 million deaths were associated with bacterial AMR in 2019 [2]. The misuse and overuse of antimicrobials in humans, animals, and plants are the main drivers of the development of drug-resistant pathogens [1]. Because antimicrobial use and resistance are interconnected across the human health, food production, animal, and environmental sectors, AMR needs to be addressed through a One Health approach.

To prevent the inappropriate use of antimicrobials in non-human sectors, particularly in livestock production, the WHO List of Medically Important Antimicrobials considers their importance in human medicine, the risk of AMR, and potential implications for human health [3]. The list is intended to assist policymakers and regulators in making risk-based decisions to minimize the adverse impact of antimicrobial use in animals on AMR in humans. All classes of antimicrobials used in animals and humans were analyzed and categorized according to their key uses in treating serious human diseases, including whether antibiotics are authorized for use in humans only or in both humans and animals. For example, carbapenems, including imipenem and meropenem, and glycopeptides, such as vancomycin, are classified as antimicrobials authorized for human use only. Despite serious restrictions on their use in food-producing animals, vancomycin-resistant and multidrug-resistant bacteria have been detected at high levels on dairy farms in Thailand [4]. Several mechanisms may contribute to resistance to multiple antimicrobial agents. Cross-resistance occurs when a single resistance mechanism reduces susceptibility to multiple agents [5]. For example, although macrolide, lincosamide, and streptogramin B (MLSB) antibiotics have distinct chemical structures, they act on overlapping ribosomal targets and may therefore exhibit cross-resistance. In contrast, co-resistance involves distinct resistance determinants within the same bacterium, which may be genetically linked on a plasmid or another mobile genetic element. Such linkage can allow exposure to one antimicrobial to select for resistance to other antimicrobials, a process referred to as co-selection.

The Clinical and Laboratory Standards Institute (CLSI) publishes the M100 document (officially titled Performance Standards for Antimicrobial Susceptibility Testing) as the global “instruction manual” and gold standard for clinical microbiology laboratories, guiding life-saving clinical decisions [6]. For bacteria isolated from animals, CLSI published the CLSI VET01S guidelines to help veterinarians determine antimicrobial susceptibility, particularly by measuring inhibition zone diameters using the Kirby–Bauer disk diffusion method [7]. However, interpretive criteria based on zone diameters for specific treatments in veterinary medicine are limited; therefore, the zone diameters defined in human guidelines are used. Even with reference to the human CLSI guidelines, the interpretive criteria do not cover all bacterial species that cause disease in animals, especially environmental pathogens associated with conditions such as mastitis. According to a Health Monitoring System and supporting evidence from recent studies by Farrell et al. [8] and Ruegg et al. [9], treatment of clinical mastitis in adult dairy cows is among the most common reasons for antimicrobial use on dairy farms. In countries where veterinary treatment practices and dairy infrastructure are still developing, such as Cambodia, a wide range of emerging bacterial pathogens has been reported, including non-aureus staphylococci associated with chronic mastitis [10]. The high infection rate and coexistence of multiple bacterial species may facilitate the horizontal transfer of antibiotic-resistance genes [11].

AMR is the capacity of bacteria to survive and proliferate despite exposure to antibiotics that would typically inhibit their growth or eliminate them. Antimicrobial resistance develops through various mechanisms, including genetic mutations, horizontal gene transfer, adaptive physiological changes, antibiotic-inactivating enzymes, target-modifying enzymes, and multidrug efflux pumps [12]. Some of these mechanisms can confer resistance to multiple antimicrobial agents, including structurally unrelated compounds, thereby contributing to cross-resistance and multidrug resistance (MDR). On farms with a high burden of infection, inappropriate antibiotic use, mixed bacterial infections, and environmental contamination with antibiotics from animal sources might accelerate the development of antibiotic resistance. Given the limitations of the available CLSI guidelines, additional interpretive approaches may be needed to help practitioners manage infections caused by emerging bacterial pathogens. Knowledge of correlations in AMR among disease-causing bacteria might help practitioners decide whether to use or avoid certain antibiotics based on their resistance relationships with other antibiotics. However, there is limited knowledge of the correlation among AMR in specific bacteria from antibiotics used on farms, antibiotics used only in humans, and other high-class antibiotics. Therefore, this study was conducted to evaluate the correlation in AMR among different antibiotics in bacteria isolated from dairy farms with heavy intramammary infections. In addition, AMR percentages were compared between a smallholder dairy farm and an intensive dairy farm in Cambodia, where the commercial dairy industry has only recently been established.

## 2. Materials and Methods

This study evaluated antimicrobial susceptibility to 17 agents, multidrug resistance (MDR), and associations among resistance phenotypes using archived bacterial isolates [13] collected from 2 dairy farms; isolates from a smallholder university training farm were excluded because of the small sample size. Briefly, Farm A was a medium-sized local farm with approximately 30 milking cows, whereas Farm B was a large intensive farm with approximately 480 purebred Holstein-Friesian milking cows. Cows at Farm A were housed on concrete floors, while those at Farm B were housed in a spacious area with a soil floor. Feed management at Farm A followed local veterinary recommendations and involved locally available roughages and mixed concentrates, whereas Farm B followed recommendations from foreign experts based on National Research Council guidelines [14]. Farm A used local animal medications, while Farm B used imported veterinary medicines. Data from interviews with farm personnel showed that the antibiotics most commonly used on both farms were long-acting penicillin in combination with streptomycin and amoxicillin for general infectious diseases and oxytetracycline for bacterial diseases and blood parasites. In addition, enrofloxacin and trimethoprim-sulfamethoxazole were used at Farm A, while gentamicin was used at Farm B. Milking at Farm A was semi-automatic and used a pipeline system, whereas Farm B used a fully automatic milking system. Farm B monitored mastitis using milk electrical conductivity, while Farm A conducted the California Mastitis Test and treated mastitis with systemic antibiotics. Average daily milk production was 10–15 kg/cow at Farm A and 15–20 kg/cow at Farm B.

Due to the use of dairy farms supplying almost all milk produced in Cambodia as a developing country building its dairy industry, a critical-case cross-sectional study was conducted from January to February 2025. Each farm was visited once, and the cows included in the study had not received antibiotic treatment for at least 1 week. All quarter milk samples from Farm A were collected, while convenience sampling was used at Farm B. At Farm A, a researcher (S.No.) and trained veterinarians aseptically collected quarter-milk samples for bacterial identification. At Farm B, an intensive dairy farm, samples were collected by trained farm staff responsible for milking and milk quality control under the guidance of the same researcher (S.No.). The samples were transported on ice within 2 h of collection to a laboratory at the Cambodia Campus of the Asian Satellite Campuses Institute of Nagoya University, located at the Royal University of Agriculture in Phnom Penh, Cambodia, for bacterial culture. Milk samples (0.01 mL) were cultured on 5% blood agar plates at 37 °C for up to 24 h. An udder quarter was considered to have an intramammary infection (IMI) when one or two pathogens were isolated at a concentration of ≥1000 colony-forming units (CFU)/ml from a single sample or at ≥100 CFU/ml from a sample collected from a clinical mastitis case [15,16]. Samples containing more than three different colony morphologies were considered contaminated and were excluded from the determination of IMI status. Colonies with different morphologies were individually subcultured on 5% blood agar plates at 37 °C for up to 24 h until pure cultures were obtained. The bacterial isolates from both farms, comprising 439 unidentified isolates, were stored at −80 °C until use.

All bacterial isolates (*n* = 439) were obtained from frozen archives and inoculated into tryptone soy broth (TSB). They were then incubated overnight at 37 °C and subsequently subcultured onto tryptone soy agar (TSA) plates. After incubation at 37 °C for 24 h, only 186 pure isolates were recovered, originating from 167 quarters and 100 cows; 19 quarters had mixed infections. The bacterial isolates were submitted to the National Medical Reference Laboratory of the National Institute of Public Health for species identification by matrix-assisted laser desorption/ionization time-of-flight mass spectrometry (MALDI-TOF MS) using the VITEK-MS (bioMérieux, Marcy-l’Étoile, France). A small portion of a fresh bacterial colony was directly deposited onto a spot on a disposable VITEK MS DS target slide using a sterile loop. Each spot was immediately overlaid with 1 µL of ready-to-use VITEK MS α-cyano-4-hydroxycinnamic acid (CHCA) matrix solution and allowed to air-dry completely at room temperature. Calibration was performed for each acquisition group using the reference strain Escherichia coli ATCC 8739. Mass spectra were acquired automatically and analyzed against the VITEK MS Knowledge Base v3.2 using the Advanced Spectra Classifier algorithm. A confidence score of 99.9% was required for species-level identification. Isolates yielding “no Identification” or multiple species choices were retested after on-target extraction with 70% formic acid, subjected to 16S rRNA gene sequencing, or reported at the genus level only.

Antimicrobial susceptibility was tested using the Kirby–Bauer disk diffusion method. Inhibition-zone diameters were interpreted using criteria from CLSI M100 version 34 and VET01S [6,7], or study-defined extrapolated thresholds, as specified in Table 1. Classifications based on the latter thresholds were designated expected antimicrobial resistance (AMR-E) and were analyzed as exploratory classifications rather than validated resistance results. The panel comprised 17 antimicrobial agents representing nine pharmacological classes and was classified according to the importance of these agents in human medicine [3]. Antimicrobials authorized for human use (HU) only included 2 classes: carbapenems (imipenem [IPM], 10 µg; meropenem [MEM], 10 µg) and glycopeptides (vancomycin [VAN], 30 µg). Highest-priority critically important antimicrobials (HPCIAs) included third-generation cephalosporins (ceftazidime [CAZ], 30 µg; cefotaxime [CTX], 30 µg; and ceftriaxone [CRO], 30 µg) and fluoroquinolones (enrofloxacin [ERFX], 5 µg; ciprofloxacin [CIP], 5 µg). Critically important antimicrobials (CIAs) included aminoglycosides (amikacin [AN], 30 µg; gentamicin [GM], 10 µg) and a macrolide (erythromycin [E], 15 µg). Highly important antimicrobials (HIAs) included an amphenicol (chloramphenicol [C], 30 µg); penicillins (ampicillin [AM], 10 µg; penicillin G [P], 10 IU; amoxicillin-clavulanic acid [AMC], 30 µg; and oxacillin [OX], 1 µg); and a potentiated sulfonamide (sulfamethoxazole-trimethoprim [SXT], 23.75/1.25 µg). Many antimicrobial agents have no established therapeutic role in bovine mastitis and may be recommended against or prohibited for use in food-producing animals in some countries. For disk diffusion testing, bacterial suspensions were prepared in sterile saline (0.85% NaCl) from overnight cultures and standardized to a 0.5 McFarland turbidity standard (approximately 1.5 × 10^8^ CFU/mL) verified using a densitometer. Suspensions were inoculated onto Mueller–Hinton agar (Himedia) prepared at a uniform depth of 4.0 ± 0.5 mm within 15 min. Antibiotic disks (Oxoid^TM^, UK) were applied to the agar surface within 15 min of inoculation. After incubation at 37 °C for 24 h, inhibition-zone diameters were measured and interpreted using the criteria specified in Table 1. For combinations assigned to AMR-E, the susceptible, intermediate, or resistant labels were operational categories based on study-defined thresholds and did not represent validated clinical susceptibility classifications.

### Statistical Analysis

Only isolate-antimicrobial combinations for which interpretive criteria were available (Table 1) were included in the analysis. To compare antimicrobial resistance between farms, the intermediate and susceptible (S + I) categories were combined into a non-resistant category, as both ‘S’ and ‘I’ predict therapeutic success, reflecting clinical treatability [17], and compared with the resistant (R) category. Differences in the proportions of AMR and AMR-E profiles between farms for each antibiotic and bacterial group were analyzed separately using Fisher’s exact test. Correlations in resistance were determined using Spearman’s correlation test, treating AMR status as ordinal data (S = 0, I = 1, and R = 2). For this study, MDR and MDR-E were operationally defined as non-susceptibility (I or R) to at least one antimicrobial agent in three or more antimicrobial classes. MDR was calculated using AMR classifications only, whereas MDR-E was calculated using an expanded panel comprising both AMR and AMR-E classifications. Species-specific intrinsic resistance was not excluded from either calculation. These study-defined classifications therefore describe observed non-susceptibility patterns and differ from the acquired-resistance framework proposed by Magiorakos et al. [18]. MDR-E additionally incorporates classifications based on unvalidated extrapolated thresholds and was treated as an exploratory index. Species-specific intrinsic resistance was not excluded from these calculations. MDR and MDR-E therefore represent study-defined classifications of observed non-susceptibility patterns and should not be interpreted as classifications restricted to acquired resistance. MDR status was defined as 0 for non-MDR and 1 for MDR. Spearman’s correlation test was also used to assess correlations between MDR status and resistance to individual antibiotics. The significance level was defined as *p* < 0.05. To account for multiple hypothesis testing across pairwise correlations, *p*-values were adjusted using the Benjamini–Hochberg false discovery rate (FDR) procedure. Correlations were considered statistically significant at an FDR-adjusted Q < 0.05. Both *p*- and Q-values were reported for exploratory analysis to generate hypotheses and identify candidate patterns for future study and for confirmatory inference, respectively.

## 3. Results

**Figure 1:**
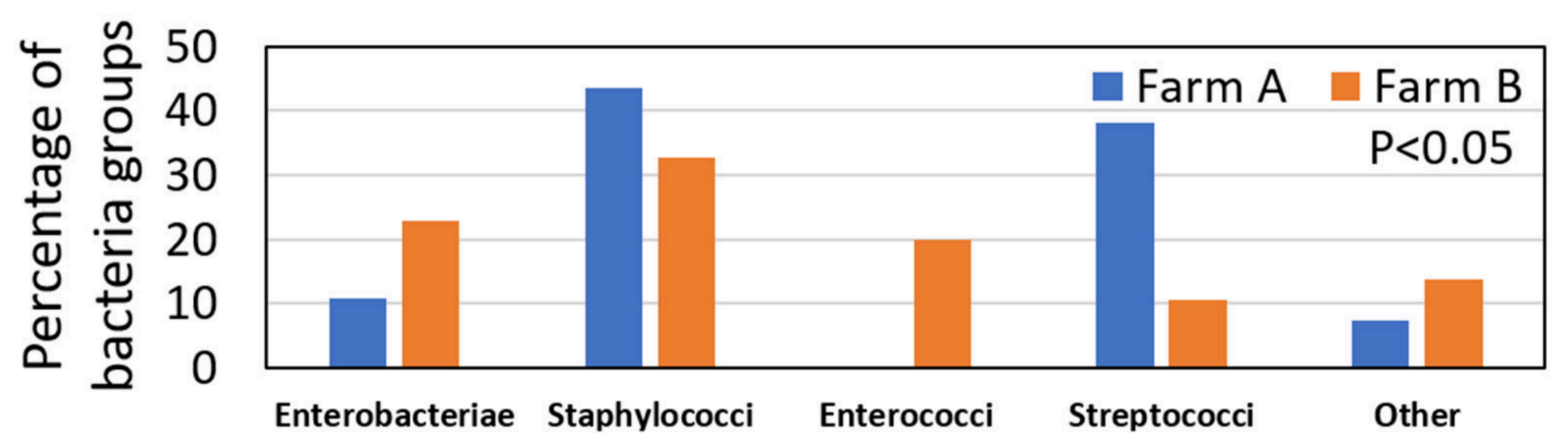
Percentages of bacteria isolated from Farm A (*n* = 55) and Farm B (*n* = 131). Fisher’s exact test was used to determine the *p*-value.

Of the 186 isolates, matrix-assisted laser desorption/ionization time-of-flight mass spectrometry (MALDI-TOF MS) analysis revealed the distribution of bacterial species isolated from the two farms (Table 2). Enterobacterales consisted primarily of *E. coli* (75%, *n* = 27), followed by *K. pneumoniae* (*n* = 4). Except for one *S. aureus* isolate, the remaining 66 staphylococcal isolates were non-aureus staphylococci. *S. chromogenes* (*n* = 43) was the most common bacterial species identified in this study. *E. hirae* (*n* = 10) was the most frequently isolated species among the enterococci. *S. uberis* (*n* = 23) was the most common bacterial species among the streptococci. Most *S. uberis* isolates were found at Farm A (*n* = 23). Three isolates of *S. gallolyticus* were found across both farms. *Lactococcus lactis* (*n* = 6) was the most common bacterium in the Other category. Comparisons of the proportions of bacterial groups between the two farms are shown in Figure 1. Non-aureus staphylococci and bacteria in the Other category were more prevalent at Farm A, whereas Farm B had higher percentages of Enterobacterales and streptococci. Enterococci were detected only at Farm B.

Antimicrobial-resistant and AMR-E bacteria, as determined from inhibition zone parameters and some modifications according to the criteria in Table 1, are shown in Table 3. Among Enterobacterales, 12 isolates were susceptible to all antimicrobials tested, while 8 were classified as multidrug-resistant. All meropenem-resistant Enterobacterales (*n* = 3) were multidrug-resistant. One staphylococcus with MDR or MDR-E was found among the 24 staphylococcal isolates from Farm A. Among the 20 *S. chromogenes* isolates from Farm A, 15 (75%) were classified as resistant to penicillin G and met the exploratory AMR-E criterion for ampicillin. In contrast, 17 (39.5%) and 31 (72.1%) of the 43 staphylococcal isolates from Farm B were MDR and MDR-E, respectively. Most MDR staphylococci were intermediate to enrofloxacin and resistant to oxacillin, while MDR-E were additionally resistant to vancomycin, erythromycin, and penicillins. Of the 26 Enterococcus isolates, 46.2% were not resistant (S and I) to all antimicrobials tested, whereas 42.3% were multidrug-resistant; most MDR isolates were either intermediate or resistant to vancomycin, erythromycin, ampicillin, and penicillin G. For streptococci, MDR for Farm A and B were 14.3% and 57.1%, respectively. All MDR streptococci were resistant to penicillin. Regarding AMR-E, all streptococci were resistant to at least one antimicrobial, with oxacillin resistance the most prevalent (88.6%). The proportions of streptococcal isolates meeting the MDR-E criterion were 52.4% at Farm A and 71.4% at Farm B. One *S. gallolyticus* isolate was resistant to six antimicrobial classes.

**Figure 2:**
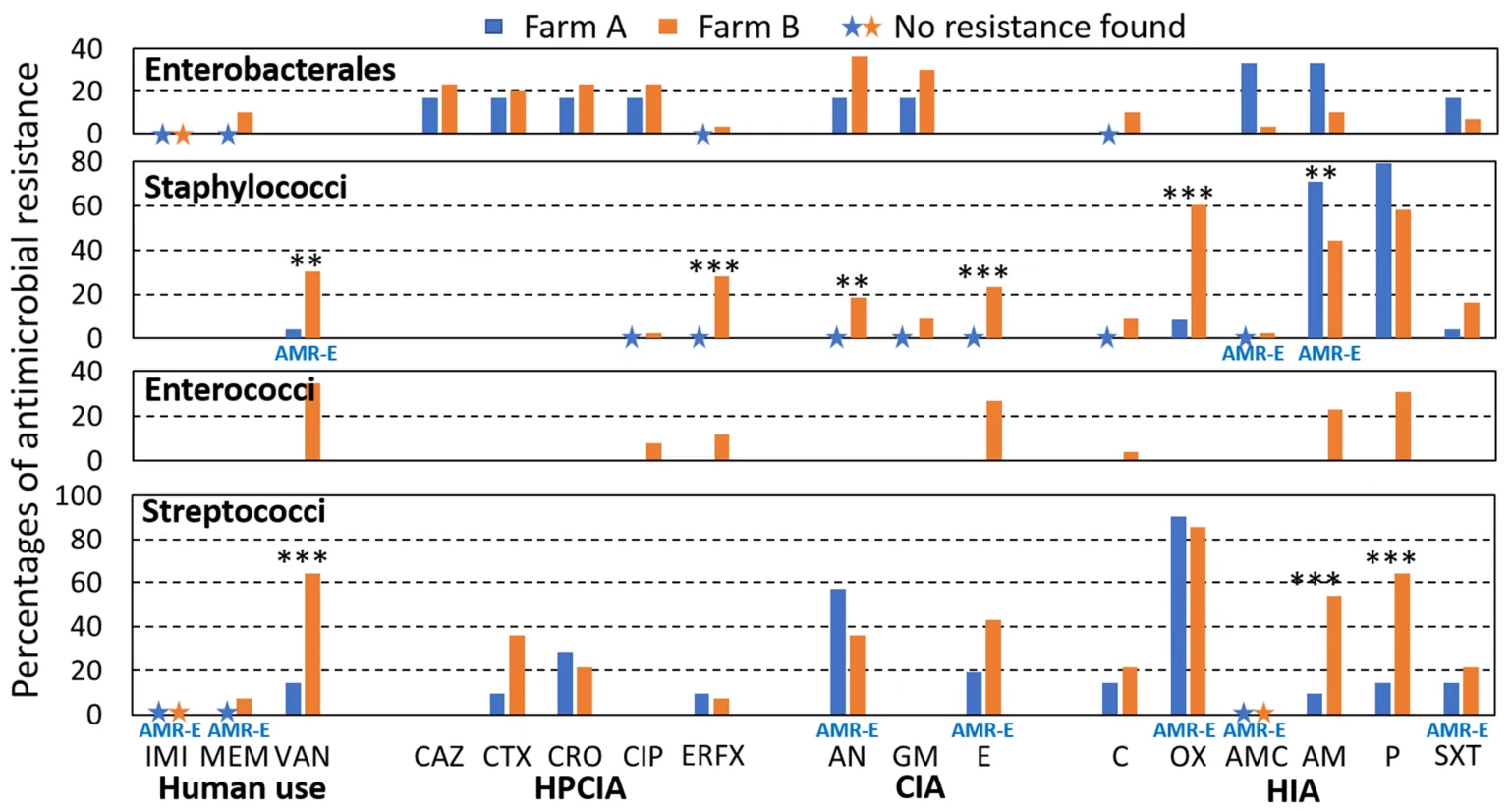
Comparison of antimicrobial resistance percentages across categories of the WHO List of Medically Important Antimicrobials [3] among bacteria isolated from milk samples collected at Farms A and B. The numbers of Enterobacterales, staphylococcal, enterococcal, and streptococcal isolates from Farms A and B were 3 and 33, 24 and 43, 0 and 26, and 21 and 14, respectively. The association between antimicrobial resistance and farm was evaluated using Fisher’s exact test. **, and *** indicate significance at *p* < 0.05, and *p* < 0.01, respectively. AMR-E denotes exploratory classifications based on study-defined extrapolated inhibition-zone thresholds (Table 1), not MIC measurements or validated clinical breakpoints.

Standard-based analyses included 13, 9, 7, and 7 agents for Enterobacterales, staphylococci, enterococci, and streptococci, respectively. The corresponding expanded exploratory panels, including additional combinations interpreted using study-defined thresholds, comprised 13, 12, 7, and 14 agents. The percentages of antimicrobial-resistant isolates from both farms are shown in Figure 2. No Enterobacterales isolates were classified as resistant to IPM. No streptococcal isolates met the study-defined AMR-E for IPM. Moderate levels of resistance to the highest-priority critically important antimicrobial (HPCIA) agents CAZ, CRO, CTX, and CIP ranged from 16.7% to 23.3% across both farms. All Enterobacterales from Farm A were susceptible to MEM, ERFX, and C, whereas all staphylococci from Farm A were susceptible to VAN, CIP, ERFX, AN, GM, E, C, and AMC. Regardless of AMR or AMR-E, among staphylococci, resistance to VAN, ERFX, AN, E, and OX at Farm B (18.6% to 60.5%) was significantly higher than at Farm A. In contrast, resistance to AM (70.8%) at Farm A was significantly higher than that at Farm B (*p* < 0.05). Enterococci were found only at Farm B, and the percentages of isolates resistant to VAN (34.6%), E (26.9%), AM (23.1%), and P (30.8%) were relatively high. Streptococci were most resistant to vancomycin (34.3%) and penicillin (34.2%). The proportions of streptococci meeting the study-defined AMR-E criterion for OX were 90.5% and 85.7% at Farms A and B, respectively. The percentages of isolates resistant to VAN, AM, and P at Farm B were significantly higher than those at Farm A (*p* < 0.05).

**Figure 3:**
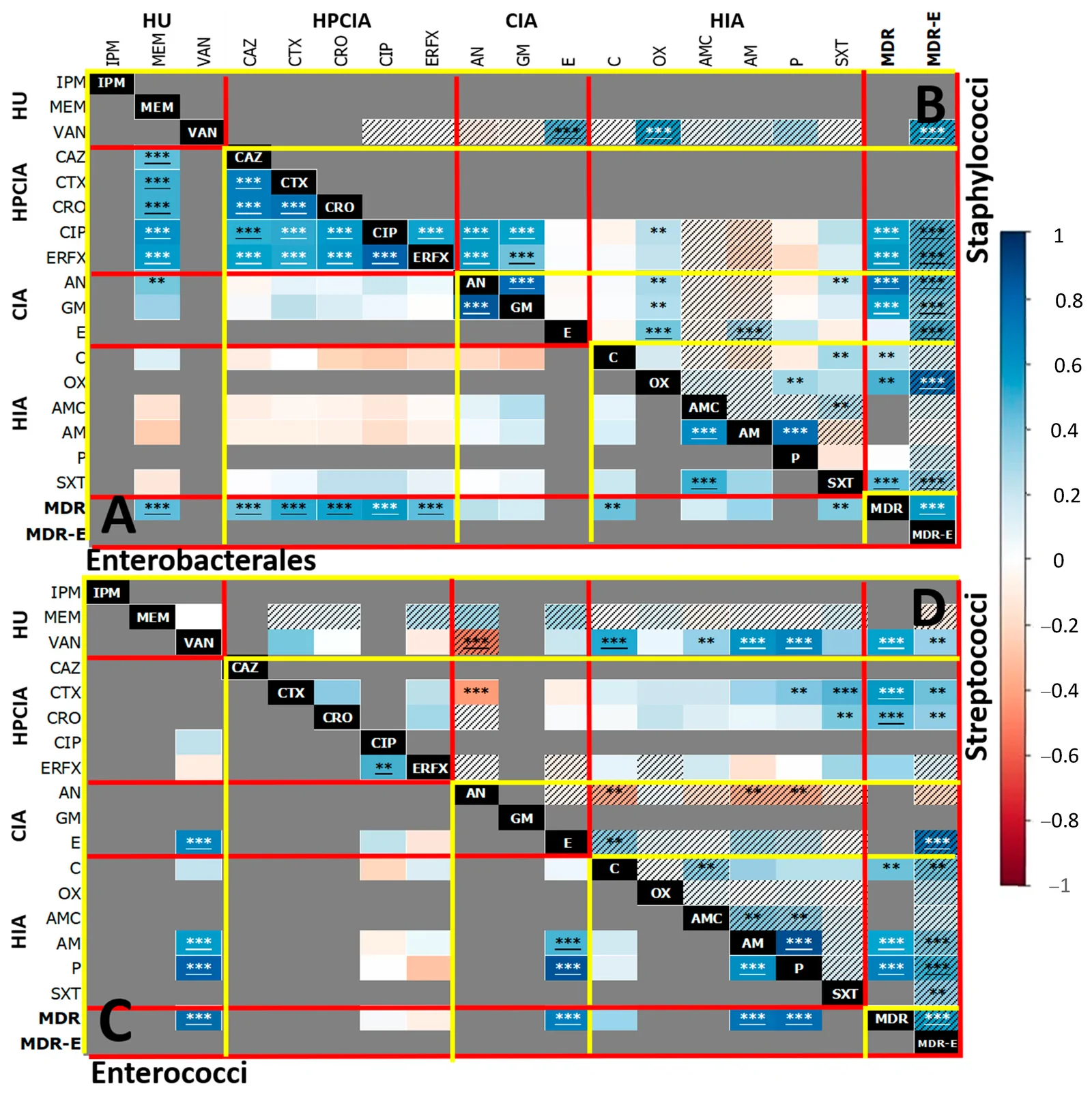
Correlations in AMR status across antibiotics based on the interpretive criteria specified in Table 1, separated by bacterial group: (**A**), Enterobacterales; (**B**), staphylococci; (**C**), enterococci; and (**D**), streptococci. Correlation coefficients are represented by the color scale shown in the bar on the right. Red and yellow lines represent the categories in the WHO List of Medically Important Antimicrobials, including those authorized for human use (HU) only, HPCIAs, CIAs, and HIAs. Antibiotics include imipenem (IPM), meropenem (MEM), vancomycin (VAN), ceftazidime (CAZ), ceftriaxone (CRO), cefotaxime (CTX), ciprofloxacin (CIP), enrofloxacin (ERFX), amikacin (AN), gentamicin (GM), erythromycin (E), chloramphenicol (C), oxacillin (OX), amoxicillin-clavulanic acid (AMC), ampicillin (AM), penicillin G (P), and sulfamethoxazole-trimethoprim (SXT). Cross-hatched cells indicate analyses involving AMR-E classifications based on study-defined extrapolated inhibition-zone thresholds. MDR and MDR-E were calculated separately as described in the Methods; MDR-E represents an exploratory index rather than validated MDR. *** and ** indicate significance nominal *p* < 0.01 and *p* < 0.05, respectively. Underlined asterisks indicate correlations with a Benjamini–Hochberg false discovery rate (FDR) at Q < 0.05. Black and white asterisks indicate correlation coefficients (ρ) < 0.5 and >0.5, respectively.

Figure 3 shows correlations in both AMR and AMR-E statuses across antibiotics according to CLSI 2024 [6] and some modifications, respectively, separated by bacterial group: A, Enterobacterales; B, staphylococci; C, enterococci; and D, streptococci. IPM was tested against Enterobacterales, and no IPM-resistant isolates were detected. For Enterobacterales, resistance to MEM, CAZ, CRO, CTX, CIP, and ERFX was highly correlated (*p* < 0.01, Q < 0.05), but showed weaker correlations with resistance to antibiotics in the critically important antimicrobial (CIA) and highly important antimicrobial (HIA). MDR was strongly correlated with antibiotic resistance to the use of human-only (HU) and HPCIA antibiotics, including MEM, CRO, CTX, CIP, and ERFX (Q < 0.05), but was weakly correlated with antibiotic resistance in the CIA and HIA groups. For staphylococci, strong correlations were found among resistance to CIP, ERFX, AN, and GM (*p* < 0.01, Q < 0.05). Interestingly, resistance to oxacillin (OX) in staphylococci was correlated with resistance to most antibiotics (*p* < 0.05), except those in the same class, including AMC and AM, in the AMR-E. In addition, resistance to AMC in staphylococci was not correlated with resistance to other antibiotics, except SXT. MDR in staphylococci was correlated with resistance to most antibiotics, including CIP, ERFX, AN, GM, and SXT (Q < 0.05). Resistance to VAN, E, AM, and P in enterococci was significantly intercorrelated and was consequently correlated with MDR. Resistance to CIP and ERFX in enterococci was correlated but showed no correlations with resistance to the other antibiotics tested. For streptococci, significant positive correlations were observed between VAN resistance and resistance to C, AM, P, and MDR (Q < 0.05). Inverse correlations were observed for VAN-AN, AN-C, AN-AM, and AN-P at nominal *p* < 0.05. MDR in streptococci was correlated with resistance to VAN, CTX, CRO, AM, and P (Q < 0.05).

## 4. Discussion

This study used 186 isolates obtained from 2 dairy farms in Cambodia, where the dairy industry is relatively new. The principal findings of this study are as follows: multidrug-resistant bacteria were detected on both farms; the bacterial composition and antimicrobial resistance profiles differed between Farms A and B; phenotypic resistance to several medically important antimicrobials was detected; and significant correlations in resistance status were observed among several antibiotics, particularly those belonging to the same class. Our previous study showed that Farm A had several mastitis-related problems, including a very high prevalence of mastitis, a high frequency of mixed bacterial infections, unusual bacteria associated with mastitis, a high prevalence of chronic mastitis, and a high prevalence of potentially contagious non-aureus staphylococci [10], with subsequent changes in milk composition [13]. Farm A had limited healthcare facilities and resources for mastitis control, whereas Farm B was an intensive dairy farm with modern equipment and expert dairy management. However, both farms had limitations in their antibiotic-use practices, particularly in their mastitis control programs. Dairy animals are considered key reservoirs of antibiotic-resistant bacteria, and the occurrence of such bacteria is closely linked to the widespread and inappropriate use of antibiotics in agriculture and veterinary medicine, particularly for mastitis treatment [19]. Poorly managed dairy bedding, manure, and processing environments can serve as reservoirs in which environmental bacteria coexist with fecal pathogens [20]. Such environments can facilitate horizontal gene transfer, allowing environmental organisms to transfer critical antimicrobial resistance genes to human and animal pathogens [21].

This study aimed to test several important antibiotics [6] not typically used in the livestock industry, including on the farms studied. These included antibiotics authorized for human use only, such as imipenem, meropenem, and vancomycin, as well as third-generation cephalosporins in the HPCIA category. To investigate antibiotic resistance on dairy farms from a One Health perspective, considering the links among humans, animals, and the environment, this study included as broad a range of antibiotic testing as possible, using modified inhibition zone criteria for combinations without applicable criteria. These modified criteria were extrapolated from other organism-antimicrobial combinations by reference to the same or a higher published minimum inhibitory concentration (MIC) breakpoints; MICs were not measured in this study. In general, breakpoints are specific to the organism–antimicrobial combination, testing method, and clinical context [6,7]. The Kirby-Bauer disk diffusion test has technical limitations; therefore, findings for combinations lacking validated disk diffusion criteria should be interpreted as preliminary exploratory observations, and broader claims should be made with caution, especially for antimicrobials authorized for human use only and HPCIAs. Due to the lack of dedicated veterinary mastitis breakpoints, reliance on human guidelines may introduce misclassification when the criteria are not applicable to the organisms or testing conditions examined. Moreover, our modified inhibition zone criteria, based on comparisons of published MIC breakpoints or, for streptococci without a validated breakpoint, on criteria intended for *S. pneumoniae*, were not validated for determining clinical resistance in the organisms examined. Therefore, our study, particularly the analyses involving AMR-E and MDR-E, was not intended to determine clinical antibiotic resistance in mastitis pathogens but rather to identify phenotypic associations among study-defined classifications across key antimicrobial classes. We retained these analyses to explore patterns involving antimicrobials for which applicable interpretive criteria were unavailable and to identify organism-antimicrobial combinations for subsequent validation. These analyses were used solely to generate hypotheses and identify candidate patterns for future study rather than for confirmatory inference. Their inclusion does not validate the extrapolated criteria, and AMR-E and MDR-E should not be interpreted as confirmed resistance or multidrug resistance. Therefore, results based on our modified inhibition zone criteria for some organism-antibiotic combinations, including staphylococci with VAN, AMC, and AM and streptococci with IPM, MEM, AN, OX, AMC, and SXT, as well as MDR-E classifications related to these combinations, must be interpreted with care. The observed frequencies and associations may depend on thresholds selected. In addition, given the limitations of these classifications, resistance requires evaluation using appropriate organism-specific susceptibility testing and applicable interpretive criteria. Specific testing, such as carbapenemase testing and detection of AMR-relevant β-lactamase genes, van genes, or mecA/mecC/PBP2a, would help characterize the underlying resistance mechanisms [22,23]. The identification of phenotypic patterns involving antimicrobials of great importance in human medicine was therefore one of the major findings of this study. However, the results must be interpreted in light of the limited availability of organism-specific veterinary breakpoints and the use of modified interpretive criteria for some organism-antimicrobial combinations. In particular, findings based on AMR-E and MDR-E cannot establish acquired resistance or support treatment recommendations.

Most bacteria used in this study were obtained from Nakamura and colleagues [13], and their species identities were confirmed using matrix-assisted laser desorption/ionization time-of-flight mass spectrometry (MALDI-TOF MS) in this study. The most common bacteria in each group were common mastitis-associated pathogens, including *E. coli* (*n* = 27, 75%) among Enterobacterales, *S. chromogenes* (*n* = 43, 64.2%) among staphylococci, and *S. uberis* (*n* = 23, 65.7%) among streptococci [24]. However, many of the bacteria identified, such as enterococci, are also found in the environment. This is also supported by our previous study, which showed that a variety of emerging bacteria might be associated with mastitis in the developing livestock industry [10]. In addition to the variety of bacteria, a variety of antimicrobial resistance phenotypes were observed. Although several of the isolated species are recognized as mastitis-associated, environmental, or opportunistic bacteria, this study was not designed to establish their etiological roles in mastitis. The bacterial composition should therefore be interpreted primarily as the composition of the isolates available for antimicrobial susceptibility testing.

Differences in bacterial distribution between Farm A and Farm B (Figure 1) may have been influenced by differences in the samples obtained and in farm management. All-quarter milk sample collection in Farm A could reflect the true AMR prevalence, but the convenience sampling design by the farm manager in Farm B might deviate from the true prevalence, either by underestimating by selecting the known healthy udder or by overestimating by selecting the tentatively mastitis quarters for evaluation. Therefore, comparisons of prevalence between farms must be carefully conducted. Farm A showed high bacterial diversity, as supported by our previous study [10]. In contrast, Farm B had higher proportions of environmental bacteria, including Enterobacterales and enterococci, which might reflect the true causes of mastitis or environmental contamination due to limitations in routine aseptic milk collection. However, our study was not intended to report the diversity of bacteria causing mastitis but rather to characterize the antibiotic resistance of bacteria obtained from dairy farms in a country with limited dairy industry infrastructure. Therefore, the isolates in this study should be referred to as mastitis pathogens with caution.

For Enterobacterales, antibiotics from all importance categories were tested (Figure 2). Among antibiotics not authorized for use on livestock farms, the human-use-only antibiotics IPM and MEM showed low levels of resistance, whereas resistance to most HPCIAs, including ceftazidime, ceftriaxone, cefotaxime, and ciprofloxacin, was moderate, ranging from 16.7% to 23.3% across both farms. In a meta-analysis, carbapenem resistance among Enterobacterales from dairy cattle remained at low levels across most countries [25]. The detection of isolates with a meropenem-resistant phenotype is of concern, as carbapenems are considered last-resort antimicrobials for treating multidrug-resistant bacteria, especially extended-spectrum β-lactamase (ESBL)-producing bacteria [26]. The relatively high rates of resistance to third-generation cephalosporins might be due to their availability in veterinary markets, especially for mastitis treatment and prevention [27]. However, resistance to amikacin and gentamicin was relatively high (>30%), especially at Farm B. This may be associated with the regular use of gentamicin at Farm B. In a human study, Gram-negative isolates collected from hospitals in Turkey exhibited high levels of resistance to gentamicin and amikacin [28], suggesting that the increased resistance could reflect both the emergence of new aminoglycoside resistance mechanisms and greater complexity among resistance mechanisms, including permeability-mediated resistance.

For staphylococci, low rates of resistance to fluoroquinolones were observed (Figure 2), whereas high rates of resistance to penicillin and ampicillin were observed on both farms, particularly at Farm A, where resistance exceeded 70%. Antibiotics, especially long-acting penicillin G–dihydrostreptomycin, have been increasingly used in Southeast Asian countries, particularly Thailand [29]. Interestingly, the resistance rates to erythromycin, oxacillin, amikacin, and enrofloxacin were significantly higher at the intensive farm (Farm B) than at the smallholder dairy farm (Farm A). Farm environments may contribute to the persistence and spread of resistant bacteria and resistance genes [30,31,32,33]. However, the present study did not quantify antimicrobial consumption, environmental antimicrobial residues, or resistance genes. Therefore, the higher resistance rates detected at Farm B cannot be directly attributed to more frequent antimicrobial use or to its intensive production system. Antibiotic resistance in enterococci was analyzed only at Farm B (Figure 2). Enterococci showed the highest rate of resistance to vancomycin (34.6%), with slightly lower rates of resistance to erythromycin, ampicillin, and penicillin. *Enterococcus gallinarum* and *E. casseliflavus*, both identified in this study, are known to exhibit intrinsic, low-level vancomycin resistance associated with chromosomal vanC genes [34]. Their inclusion may therefore contribute to the pooled enterococcal vancomycin results, which should not be interpreted as an estimate of acquired vancomycin resistance. Because MIC testing and resistance gene characterization were not performed, the present study cannot distinguish the contribution of intrinsic resistance from that of acquired resistance determinants. In contrast, our previous study using enterococci from smallholder dairy farms in Cambodia did not identify any vancomycin-resistant enterococci [22]. Enterococci typically live harmlessly in the human intestines and genital tract, but vancomycin-resistant enterococci can cause severe infections, such as bloodstream, urinary tract, and wound infections, particularly in vulnerable individuals [35]. Therefore, confirmation of vancomycin resistance by broth microdilution, together with detection of relevant resistance determinants, including vancomycin-resistance genes, is necessary before these isolates can be definitively classified as vancomycin-resistant enterococci.

More than 80% of streptococci from both farms met the study-defined AMR-E criterion for oxacillin, whereas a much lower proportion were classified as resistant to penicillin (Figure 2). Because the oxacillin criterion was extrapolated and was not validated for the species examined, this finding should not be interpreted as evidence of acquired oxacillin resistance. Interestingly, Farm B had higher rates of isolates classified as resistant to vancomycin and chloramphenicol, neither of which is authorized for use in livestock, than Farm A. High rates of resistance among streptococci isolated from mastitis cases have also been reported using disk diffusion techniques: 34% [36] in Korea, up to 73.2% for oxacillin in Switzerland [37], and 54.8% for vancomycin among unidentified streptococci in Thailand [4]. However, direct comparisons with these reports are limited by potential differences in bacterial species, testing conditions, and interpretive criteria, and these reports do not validate the extrapolated thresholds used in this study. The routine use of intramammary β-lactam infusions (such as cloxacillin) across entire herds during the drying-off period to treat or prevent subclinical mastitis may act as a selective pressure on environmental and contagious streptococcal strains [38]. However, the present study cannot establish a relationship between such exposure and the high proportion of isolates meeting the AMR-E criterion for oxacillin. The difference in vancomycin classifications between the two farms may have reflected differences in bacterial compositions. Further testing using species-appropriate methods and applicable interpretive criteria is needed to clarify these findings.

Different correlations between meropenem resistance and resistance to HPCIAs were observed in Enterobacterales, which showed significant correlations, and streptococci, which showed no correlations. Our study showed that meropenem resistance was significantly correlated with resistance to third-generation cephalosporins and aminoglycosides and was also correlated to some extent with resistance to other antibiotics. The few meropenem-resistant Enterobacterales (*n* = 3), referred to as Carbapenem-Resistant Enterobacterales (CRE), were classic examples of multidrug-resistant organisms [39]. This study confirmed that these CRE were resistant to more than three antibiotic classes (Table 3). Organisms that carry a carbapenemase gene are often resistant to multiple drug classes due to the co-location of multiple antibiotic resistance determinants on plasmids that are transferred among bacteria [40]. Carbapenem-resistant Enterobacterales have frequently shown resistance to third-generation cephalosporins in humans [41] and on small-scale poultry farms [42]. In a previous study, carbapenem resistance was primarily mediated by blaNDM-5, carried on species-specific plasmids: IncFIA/IncFII in *E. coli* and IncFIB/IncHIB megaplasmids in *K. pneumoniae*, frequently co-harboring additional aminoglycoside, sulfonamide, and fluoroquinolone resistance genes [43]. No correlations were observed between resistance to carbapenems or third-generation cephalosporins and resistance to chloramphenicol, penicillins, or sulfamethoxazole-trimethoprim. The lack of correlation between meropenem resistance and resistance to other antibiotics might be due to the presence of only one meropenem-resistant streptococcal isolate (Table 3). However, carbapenem-resistant streptococci have been reported as rare multidrug-resistant bacteria [44].

Vancomycin resistance was correlated with resistance to erythromycin and oxacillin in staphylococci; erythromycin, ampicillin, and penicillin in enterococci; and ceftriaxone, chloramphenicol, amoxicillin-clavulanic acid, ampicillin, penicillin, and sulfamethoxazole-trimethoprim in streptococci (Figure 3). Resistance to both oxacillin and vancomycin has also been reported in dogs [45]. Vancomycin is a glycopeptide that targets bacterial cell wall synthesis by binding to D-Ala-D-Ala precursors—a mechanism entirely distinct from those of macrolides, β-lactams, or antifolates [46]. Therefore, a positive correlation does not mean that vancomycin shares a resistance mechanism with these other drugs. Although vancomycin was not used on the farms, the observed correlations may reflect co-selection or genetic linkage of different resistance determinants [47].

As shown in Figure 3, resistance to most antibiotics within the same class was correlated, including resistance to cephalosporins, fluoroquinolones, aminoglycosides, and penicillins. These correlations indicate phenotypic associations but do not establish a shared resistance mechanism. Antibiotics within the same class may share related chemical structures, mechanisms of action, and cellular targets. A single resistance mechanism may confer reduced susceptibility to multiple drugs within the same class; this phenomenon is termed cross-resistance. For example, chromosomal mutations affecting DNA gyrase can reduce susceptibility to multiple fluoroquinolones [48]. However, whether cross-resistance accounts for the associations observed in this study remains to be determined. Interestingly, negative correlations were observed between amikacin resistance and resistance to several other antimicrobials, including vancomycin, cefotaxime, chloramphenicol, ampicillin, and penicillin (Figure 3). A negative correlation in an AMR correlation matrix indicates an inverse relationship: bacterial isolates that develop or possess resistance to amikacin were statistically more likely to be susceptible to vancomycin, cefotaxime, chloramphenicol, ampicillin, and penicillin, and vice versa. Such an inverse relationship may be compatible with collateral sensitivity, in which resistance to one antibiotic is associated with increased susceptibility to another [49]. However, the negative phenotypic correlations observed here cannot by themselves demonstrate collateral sensitivity; this interpretation requires confirmation using individual strains under controlled experimental conditions.

## 5. Conclusions

In conclusion, multidrug-resistant bacteria were detected among isolates obtained from two dairy farms in Cambodia, highlighting the need for antimicrobial resistance surveillance as the country’s dairy industry continues to develop. Antimicrobial susceptibility profiles differed between the two farms, with the direction and magnitude of these differences varying by bacterial group and antimicrobial agent. These findings describe the analyzed isolates rather than farm-level resistance prevalence. Significant correlations in phenotypic susceptibility classifications were observed across several antimicrobials, particularly between agents within the same class. These patterns represent phenotypic associations among responses to medically important antimicrobials but do not establish cross-resistance or co-resistance, because the underlying resistance mechanisms were not investigated in this study. AMR-E and MDR-E results were based on unvalidated extrapolated thresholds and should be regarded as hypothesis-generating rather than evidence of confirmed resistance or multidrug resistance. However, given the limitations of the testing methods and interpretive criteria used, and the limited information available on antimicrobial resistance in many of the bacterial species identified in this study, further studies using appropriate susceptibility testing, including minimum inhibitory concentration determination where applicable, together with molecular characterization, are needed to validate these observations and investigate the underlying resistance mechanisms.

## Figures and Tables

**Table 1:** List of antibiotics and their classes with inhibition-zone diameter criteria used for standard-based and exploratory classifications. Criteria were obtained from CLSI M100 version 34 [6] and VET01S [7] or extrapolated for this study. Extrapolated criteria are identified as expected antimicrobial resistance (AMR-E) and are not validated clinical breakpoints for the corresponding organism-antimicrobial combinations. Information on Critically Important Antimicrobials for Human Medicine [3] included use in humans (HU) only, highest priority critically important antimicrobials (HPCIA), critically important antimicrobials (CIAs), and highly important antimicrobials (HIAs). Inhibition zone diameters are indicated as >value for susceptible/ranges for intermediate/<value for resistant.

Antibiotic Class	Type ^1^	Important Class	Enterobacterales	Staphylococci	Enterococci	Streptococci
Carbapenem	IPM	HU	23/20–22/19 ^2,3^	-	--	23/20–22/19 ^10,11^
	MEM	HU	23/20–22/19 ^2^	-	-	23/20–22/19 ^10,11^
Glycopeptide	VAN	HU	-	17/15–16/14 ^10,11^	17/15–16/14 ^5^	17/-/- ^7^
Cephalosporin	CAZ	HPCIA	21/18–20/17 ^2^	-	-	-
	CTX	HPCIA	23/20–22/19 ^2^	-	-	24/-/- ^7^
	CRO	HPCIA	26/23–25/22 ^2^	-	-	24/-/-- ^7^
Fluoroquinolone	CIP	HPCIA	26/22–25/21 ^2^	21/16–20/15 ^4,5^		-
	ERFX	HPCIA	23/17–22/16 ^9^			
Aminoglycoside	AN	CIA	20/17–19/16 ^2^	17/15–16/14 ^9^	-	20/17–19/16 ^10,11^
	GM	CIA	18/15–17/14 ^2^	15/13–14/12 ^4,8^	-	-
Macrolide	E	CIA	-	23/14–22/13 ^4,5,8^		21/16–20/15 ^6,11^
Chloramphenicol	C	HIA	18/13–17/12 ^2,4,5,8^			21/18–20/17 ^7^
Penicillin	OX	HIA	-	18/-/17 ^3^	-	20/-/- ^6,11^
	AMC	HIA	18/14–17/13 ^2,8^	18/14–17/13 ^10,11^	-	18/14–17/13 ^10,11^
	AM	HIA	17/14–16/13 ^2,8^	24/-/- ^10,11^	17/-/16 ^5^	24/-/- ^7^
	P	HIA	-	29/-/28 ^4^	15/-/14 ^5^	24/-/- ^7^
Potentiated sulfonamides	SXT	HIA	16/11–15/10 ^2,4,8^		-	19/16-18/15 ^6,11^

^1.^ The antimicrobial agents and disk contents were as follows: imipenem 10 µg (IPM), meropenem 10 µg(MEM), vancomycin 30 µg (VAN), ceftazidime 30 µg (CAZ), cefotaxime 30 µg (CTX), ceftriaxone 30 µg(CRO), ciprofloxacin 5 µg (CIP), enrofloxacin 5 µg (ERFX), amikacin 30 µg (AN), gentamicin 10 µg (GM), erythromycin 15 µg(E), chloramphenicol 30 µg (C), oxacillin 1 µg(OX), amoxicillin-clavulanic acid 30 µg (AMC), ampicillin 10 µg (AM), penicillin G 10 IU (P), and sulfamethoxazole-trimethoprim 23.75/1.25 µg (SXT). ^2–7^ Data were obtained from CLSI M100 [7] for the following bacterial groups: ^2^ Enterobacterales except Salmonella/Shigella, ^3^ *S. epidermidis*, ^4^ all staphylococci, ^5^ enterococci, ^6^ *S. pneumoniae*, and ^7^ Streptococci with beta-hemolysis, respectively. ^8,9^ Data were obtained from CLSI VET01S [8]: ^8^ human and ^9^ animal. ^10.^ Study-defined inhibition-zone thresholds were extrapolated from other organism-antimicrobial combinations by reference to published MIC breakpoints. This extrapolation was not validated, and MICs were not measured in the present study. ^11.^ These thresholds were used only for exploratory AMR-E classifications and should not be interpreted as validated resistance criteria.

**Table 2:** Distribution of bacterial species among bacterial groups categorized according to CLSI criteria. Bacterial isolates were obtained from milk samples collected from 2 dairy farms in Cambodia. Isolates assigned to the “Other” category were excluded from subsequent antimicrobial susceptibility analysis.

Bacterial Group	Bacterial Species
Enterobacterales (*n* = 36)	*Citrobacter koseri* (*n* = 2), *Enterobacter hormaechei* (*n* = 1), *Enterobacter kobei* (*n* = 2), *Escherichia coli* (*n* = 27), *Klebsiella pneumoniae*(*n* = 4)
Staphylococci (*n* = 67)	*S. chromogenes* (*n* = 43), *S. cohnii ssp cohnii* (*n* = 1), *S. haemolyticus* (*n* = 5), *S. pseudintermedius* (*n* = 1), *S. sciuri* (*n* = 14), *S. simulans* (*n* = 1), *S. xylosus* (*n* = 1), *S. aureus* (*n* = 1)
Enterococci (*n* = 26)	*E. faecalis* (*n* = 6), *E. faecium* (*n* = 3), *E. gallinarum* (*n* = 5), *E. hirae* (*n* = 10), *E. mundtii* (*n* = 1), *E. casseliflavus* (*n* = 1)
Streptococci (*n* = 35)	*S. dysgalactiae ssp dysgalactiae* (*n* = 1), *S. equinus* (*n* = 3), *S. gallolyticus ssp gallolyticus* (*n* = 2), *S. gallolyticus ssp pasteurinanus* (*n* = 1), *S. infantarius ssp infantarius* (*n* = 2), *S. parauberis* (*n* = 3), *S. uberis* (*n* = 23),
Other (*n* = 22)	*Acinetobacter baumannii* (*n* = 2), *A. calcoaceticus* (*n* = 1); *Aerococcus viridans* (*n* = 2); *Bacillus cereus* (*n* = 3); *B. megaterium* (*n* = 1); *Corynebacterium amycolatum* (*n* = 4); *Geobacillus thermoleovorans* (*n* = 1); *Lactococcus lactis* (*n* = 6); *Macrococcus caseolyticus* (*n* = 1); *Weissella confusa* (*n* = 1)

**Table 3:** Antimicrobial-resistant (AMR) and AMR-E (the gray highlighted area) bacteria were determined according to the interpretive criteria specified in Table 1 and used to determine multidrug resistance (MDR) and expected MDR (MDR-E; the gray highlighted area), as non-susceptible (I and R) to at least 1 antimicrobial agent in 3 or more antimicrobial classes of AMR and AMR-E, respectively. S, I, and R indicate susceptible, intermediate, and resistant, respectively. Black and white squares indicate MDR and non-MDR isolates, respectively. The antimicrobial classes are as follows: CAR, carbapenems; CEP, third-generation cephalosporins; FLU, fluoroquinolones; AMI, aminoglycosides; and PEN, penicillins.

CIA-HM			HU			HPCIA					CIA			HIA						AMR Class (n)	MDR	MDR-E
AM Class			CAR		VAN	CEP			FLU		AMI		E	C	PEN				SXT			
Group	Farm	Species	IPM	MEM		CAZ	CTX	CRO	CIP	ERFX	AN	GM			OX	AMC	AM	P				
**Enterobacterales**	**Farm A**	**CK**	S	S		R	R	R	S	S	S	I		S		R	R		R	4	■	
		**EH**	S	S		S	S	S	S	S	I	I		S		S	S		S	1	□	
		***E. coli***	S	S		S	I	S	S	S	I	I		S		R	R		S	3	■	
			S	S		S	R	S	R	I	S	S		S		S	S		S	2	□	
			I	S		S	S	S	S	S	R	R		S		S	S		S	1	□	
		**KP**	S	S		I	S	S	S	S	S	S		S		S	S		S	1	□	
	**Farm B**	**CK**	S	S		R	R	R	I	I	I	I		S		S	R		S	4	■	
		**EK**	S	S		I	S	S	I	I	I	S		S		S	S		S	3	■	
			I	R		R	R	R	R	I	R	R		S		S	S		S	3	■	
		***E. coli***	S	R		S	I	S	R	I	I	S		R		S	S		S	4	■	
			S	S		I	I	I	I	S	S	S		R		S	S		S	3	■	
			S	S		S	S	S	S	S	S	S		S		S	S		S	0	□	
			S	S		S	S	S	S	S	S	S		S		S	S		S	0	□	
			S	S		S	R	S	R	S	R	I		S		S	S		R	4	■	
			S	S		R	R	S	I	I	S	S		S		S	S		S	2	□	
			S	S		I	S	S	I	S	S	S		S		S	S		S	2	□	
			S	R		R	R	R	R	I	R	R		S		S	S		S	3	■	
			S	S		S	S	S	S	S	R	R		S		I	I		S	2	□	
			S	S		S	S	S	S	S	I	I		S		S	S		S	1	□	
			I	S		S	S	S	S	S	R	I		S		S	I		S	2	□	
			S	S		S	S	S	S	S	I	R		S		S	S		S	1	□	
			I	S		S	I	S	R	R	I	I		I		R	R		R	6	■	
			S	I		R	R	R	R	I	R	R		S		S	S		S	3	■	
			S	S		S	S	S	S	S	R	R		S		S	S		S	1	□	
			S	S		S	S	S	I	S	R	R		S		S	I		S	3	■	
			S	S		I	I	S	S	S	I	I		S		S	I		S	3	■	
			S	S		S	S	S	S	S	R	R		S		I	I		S	2	□	
			S	S		S	S	S	S	S	I	I		S		S	S		S	1	□	
			S	S		S	S	S	S	S	I	I		S		S	S		S	1	□	
			S	S		S	R	S	S	S	S	S		S		S	S		S	1	□	
			S	S		S	I	S	S	S	S	S		R		S	I		S	3	■	
			S	S		S	S	S	S	S	S	S		S		S	S		S	0	□	
			S	S		S	S	S	S	S	S	S		S		S	R		S	1	□	
		**KP**	S	S		S	I	S	S	S	R	I		S		S	S		S	2	□	
			S	S		R	R	I	I	I	S	S		S		S	S		S	2	□	
			S	I		R	R	R	R	I	R	R		S		S	S		S	3	■	
**Staphylococci**	**Farm A**	***S. chromogenes***			S				S	S	S	S	S	S	S	S	R	R	S	0	□	□
					S				S	S	S	S	S	S	S	S	R	R	S	0	□	□
					S				S	S	S	S	I	S	S	S	R	R	S	1	□	□
					I				S	S	S	S	S	S	S	S	R	R	S	0	□	□
					S				S	S	S	S	S	S	S	S	R	R	S	0	□	□
					S				S	S	S	S	I	S	S	S	R	R	S	1	□	□
					S				S	S	S	S	S	S	S	S	R	R	S	0	□	□
					S				S	S	S	S	S	S	S	S	R	R	S	0	□	□
					R				S	S	S	S	S	S	S	S	R	R	S	0	□	□
					S				S	S	S	S	S	S	S	S	R	R	S	0	□	□
					S				S	S	S	S	S	S	S	S	R	R	S	0	□	□
					S				S	S	S	S	S	S	S	S	R	R	S	0	□	□
					S				S	S	S	S	S	S	S	S	R	R	S	0	□	□
					S				S	S	S	S	S	S	S	S	S	S	S	0	□	□
					I				S	S	S	S	S	S	R	S	S	S	S	1	□	□
					S				S	S	S	S	S	S	S	S	S	R	S	0	□	□
					S				S	S	S	S	S	S	S	S	R	R	S	0	□	□
					S				S	S	S	S	S	S	S	S	S	S	S	0	□	□
					S				S	S	S	S	S	S	S	S	R	R	S	0	□	□
					S				S	S	S	S	S	S	S	S	S	R	S	0	□	□
		**SH**			S				S	S	S	S	S	I	R	S	R	R	R	3	□	■
					S				S	S	S	S	S	S	S	S	S	S	S	0	□	□
					S				S	S	S	S	S	S	S	S	S	S	S	0	□	□
		**SP**			S				S	S	S	S	S	S	S	S	R	R	S	0	□	□
	**Farm B**	**SA**			R				I	R	S	S	R	S	R	S	R	R	S	3	□	■
		***S. chromogenes***			I				S	I	S	S	I	S	S	S	S	S	S	2	□	■
					I				S	I	S	S	S	R	S	S	S	S	S	2	□	■
					S				S	S	S	S	S	R	S	S	S	S	S	1	□	□
					R				S	I	I	S	I	S	R	S	R	R	I	5	□	■
					R				S	S	R	R	R	S	R	S	R	R	S	3	□	■
					S				I	R	R	I	S	S	R	S	R	R	S	3	□	■
					I				S	S	S	S	S	S	S	S	S	S	S	0	□	□
					R				S	S	S	S	S	I	R	S	R	R	I	3	□	■
					S				I	I	I	I	I	S	R	S	S	S	S	4	□	■
					R				S	S	S	S	R	S	R	S	R	R	S	2	□	■
					S				S	S	S	S	S	R	S	S	S	S	S	1	□	□
					S				S	I	S	S	S	S	R	S	S	S	S	2	□	□
					S				S	S	S	S	S	S	S	S	S	S	S	0	□	□
					S				I	I	S	S	R	S	S	S	R	R	S	2	□	■
					S				I	I	R	R	S	S	S	S	S	S	I	3	□	■
					S				I	I	R	S	S	S	R	S	S	S	R	4	□	■
					R				S	S	S	S	I	S	R	S	R	R	S	2	□	■
					S				I	I	R	I	S	S	R	S	R	R	S	3	□	■
					S				S	S	S	S	S	S	S	S	R	R	S	0	□	□
					S				S	I	I	S	S	S	R	S	R	R	S	3	□	■
					S				S	S	S	S	S	S	S	S	R	R	S	0	□	□
					I				S	S	S	S	S	S	R	R	R	R	R	2	□	■
					R				S	S	S	S	R	S	R	S	R	R	S	2	□	■
**Staphylococci**	**Farm B**	**SC**			R				S	S	S	S	R	S	R	S	R	R	S	2	□	■
		**SH**			R				S	S	S	S	S	S	R	S	S	R	R	2	□	■
					S				S	S	S		S	S	S	S	S	S	R	1	□	□
		***S. sciuri***			I				I	I	I	I	I	R	R	I	I	I	I	4	■	■
					S				S	S	S	S	S	S	S	S	S	S	R	1	□	□
					I				R	I	R	R	S	I	R	S	S	R	I	6	■	■
					S				I	I	I	S	S	S	S	S	S	S	I	4	■	■
					S				S	I	R	R	S	I	R	S	S	R	S	5	■	■
					S				I	I	R	I	S	S	S	S	S	S	R	4	■	■
					I				S	S	S	S	R	I	R	S	R	R	R	4	■	■
					S				S	S	S	S	S	S	S	S	S	S	S	0	□	□
					R				S	S	S		R	S	R	S	R	R	S	2	□	■
					I				I	S	S		S	S	R	S	S	R	S	2	□	■
					S				S	S	I	S	I	S	S	S	S	S	S	3	□	□
					R				I	I	I	I	R	I	R	I	R	R	I	2	□	■
					I				S	I	S	S	S	S	R	S	S	R	S	2	□	■
					R				I	S	S		S	S	R	S	S	R	S	2	□	■
		**SS**			I				S	I	S		S	S	S	S	S	S	S	1	□	□
		**SX**			R				S	S	S	S	R	S	R	S	R	R	S	2	□	■
**Enterococci**	**Farm B**	***E. faecalis***			S				I	I			S	S			R	S		2	□	
					I				S	I			S	S			R	S		3	■	
					S				S	S			S	S			S	S		0	□	
					I				S	I			I	S			S	S		1	□	
					R				R	S			R	S			R	R		4	■	
					S				S	S			S	S			S	S		0	□	
		**EF**			I				R	R			I	S			S	S		3	■	
					S				I	I			I	S			S	S		2	□	
					R				S	S			R	S			R	R		3	■	
		***E. gallinarum***			I				S	I			S	R			S	S		3	■	
					S				S	I			S	S			S	S		1	□	
					I				I	I			S	S			S	S		2	□	
					S				S	I			S	S			S	S		1	□	
					S				S	S			S	S			S	S		0	□	
		***E. hirae***			S				S	S			I	S			S	S		1	□	
					R				S	S			R	S			R	R		3	■	
					I				I	I			S	S			S	S		2	□	
					R				S	I			R	I			R	R		5	■	
					R				I	I			R	S			R	R		4	■	
					R				I	I			I	I			R	R		3	■	
					R				S	S			R	S			R	R		3	■	
					R				I	I			S	S			S	S		2	□	
					S				S	I			S	S			S	S		1	□	
					R				I	R			R	S			R	R		4	■	
		**EM**			I				I	I			I	I			I	I		1	□	
		**EC**			S				I	R			I	S			S	S		2	□	
**Streptococci**	**Farm A**	**SG**	S	S	S		S	S		I	R		I	I	R	S	S	S	I	3	□	■
		**SP**	S	S	S		S	S		S	R		S	S	S	S	S	S	S	0	□	□
		***S. uberis***	S	S	R		R	R		I	S		I	S	R	S	S	S	R	4	■	■
			S	S	S		S	R		S	R		S	S	R	S	S	S	S	1	□	□
			I	I	I		I	R		I	R		I	I	R	I	I	R	I	2	■	■
			S	S	S		R	S		S	I		I	S	R	S	S	S	S	1	□	■
			S	S	S		S	R		R	I		R	R	R	S	S	S	S	3	■	■
			S	S	S		S	S		I	R		S	S	R	S	S	S	S	1	□	□
			S	S	S		S	S		S	S		R	S	S	S	R	R	S	1	□	■
			I	I	I		I	I		I	I		I	I	R	I	I	I	I	0	□	□
			S	S	S		S	S		S	I		S	S	R	S	S	S	S	0	□	□
			S	S	S		S	R		S	R		R	S	R	S	S	S	R	2	□	■
			S	S	S		S	S		S	R		I	S	R	S	S	S	I	0	□	□
			S	S	R		S	S		S	S		I	R	R	S	S	S	R	3	□	■
			S	I	S		S	S		S	R		S	S	R	S	S	S	S	0	□	□
			S	S	S		S	R		S	R		I	S	R	S	S	S	S	1	□	■
			S	S	S		S	S		S	R		S	S	R	S	S	S	S	0	□	□
			S	S	S		S	S		S	R		I	S	R	S	S	S	S	0	□	■
			S	S	S		S	S		S	R		S	S	R	S	S	S	S	0	□	□
			S	S	S		S	S		R	I		R	R	R	S	S	S	S	2	□	■
			S	S	R		S	S		S	S		S	S	R	S	R	R	S	2	□	□
	**Farm B**	**SD**	S	S	S		S	S		S	R		R	S	R	S	S	S	S	0	□	■
		**SE**	S	S	S		R	R		I	S		S	S	R	S	R	R	I	4	■	■
			S	S	R		S	S		S	R		R	S	R	S	R	R	S	2	□	■
			S	S	R		R	S		I	S		S	S	R	S	S	R	I	4	■	■
		**SG**	S	R	R		R	R		R	R		S	I	R	S	R	R	R	6	■	■
			S	S	S		S	S		I	I		S	S	R	S	S	S	S	1	□	□
		**SI**	S	S	S		S	S		I	R		S	S	S	S	S	S	R	2	□	□
			S	S	R		R	S		S	S		R	R	R	S	R	R	I	4	■	■
		**SPA**	S	I	S		S	S		I	R		S	S	R	S	S	S	S	1	□	□
			S	S	R		S	S		S	I		R	I	R	S	R	R	S	3	■	■
		***S. uberis***	S	S	R		R	R		S	S		S	R	R	I	R	R	R	5	■	■
			S	S	R		S	S		S	S		S	R	S	S	S	S	S	2	□	□
			S	S	R		S	S		S	I		R	I	R	I	R	R	S	3	■	■
			S	S	R		S	S		S	I		R	I	R	S	R	R	I	4	■	■

CK (*C. koseri*), EH (*E. hormaechei*), EK (*E. kobei*), KP (*K. pneumoniae*), SC (*S. cohnii ssp cohnii*), SH (*S. haemolyticus*), SP (*S. pseudintermedius*), SS (*S. simulans*), SX (*S. xylosus*), SA (*S. aureus*), EF (*E. faecium*), EM (*E. mundtii*), EC (*E. casseliflavus*), SD (*S. dysgalactiae*), SE (*S. equinus*), SG (*S. gallolyticus*), SI (*S. infantarius*), SPA (*S. parauberis*).

## Data Availability

The data presented in this study are available on request from the corresponding authors.
